# Patient perspectives on having multiple versus single prescribers of chronic disease medications: results of a qualitative study in a veteran population

**DOI:** 10.1186/s12913-014-0490-8

**Published:** 2014-10-25

**Authors:** Corrine I Voils, Betsy Sleath, Matthew L Maciejewski

**Affiliations:** Department of Veterans Affairs, Center for Health Services Research in Primary Care, Durham VAMC, 508 Fulton St. (152), Durham, NC 27705 USA; Division of General Internal Medicine, Department of Medicine, Duke University Medical Center, Durham, NC USA; Division of Pharmaceutical Outcomes and Policy, UNC Eshelman School of Pharmacy, University of North Carolina, Chapel Hill, NC USA

**Keywords:** Comorbidity, Multi-morbidity, Complex patient, Prescriber, Provider, Veterans

## Abstract

**Background:**

Patients with multiple chronic conditions often have multiple prescribers, which has been associated with greater health care utilization and medication nonadherence in claims-based analyses. This qualitative study was conducted to understand the reasons why patients have increasing numbers of prescribers of medications and to understand patient perspectives on advantages and disadvantages of having multiple prescribers, including effects on medication supply.

**Methods:**

This qualitative study involved three focus groups comprising 23 outpatients from a single Veterans Affairs (VA) Medical Center with at least one chronic cardiometabolic condition (hypertension, diabetes, dyslipidemia, or congestive heart failure). Participants were asked about their experiences, including perceived of advantages and disadvantages, of having multiple prescribers of cardiometabolic medications. Conventional content analysis was used to analyze the data.

**Results:**

Multiple prescribers arose through referrals and patients actively seeking non-VA prescribers (primary care and/or specialist) to maximize timeliness and access to medications, provide access to medications not on the VA formulary, and minimize out-of-pocket costs. Patients seeking non-VA care had to coordinate own their care by sharing prescriptions and test results to their prescribers within and outside VA.

**Conclusions:**

Prescribing physicians should engage in open dialogue with patients to create a shared understanding of patient and provider goals and priorities for chronic disease medications.

## Background

Patients with multiple chronic conditions (MCC) often have complex medication regimens, which can increase their risk for medication side effects or interactions. Additionally, MCC patients typically see multiple providers [1] and prescribers [2], which can complicate care coordination and medication management [3,4]. Medication management and medication reconciliation in the context of hospital discharge has been studied extensively. Less is known about coordination of medications in the outpatient setting. Care coordination, medication coordination, and regimen optimization may be optimal if patients obtained outpatient care from a single provider or single care team [5]. Recently, we found that veterans with one VA prescriber of cardiometabolic medications in a single Veterans Affairs (VA) medical center had higher medication refill adherence [6] and lower emergency department and hospital utilization than veterans with multiple prescribers [2].

Implicit in these VA claims analyses is the assumption that patients are passive recipients of multiple prescribers, such as when a patient is referred to a new prescriber (e.g., primary care physician requests a specialist consult). However, some patients may actively seek additional prescribers for various reasons (e.g., dissatisfaction with current prescriber, desire for particular medications, drug diversion). Veterans are an appropriate patient population in which to examine these issues because many veterans have multiple conditions [7,8] and they can obtain care in different health systems (i.e., solely VA care in an integrated health care system with a comprehensive electronic health record (EHR); non-VA care; or a combination of VA and non-VA care) [9]. Regimen optimization is likely to be particularly challenging for veterans who obtain care from multiple prescribers across unaffiliated health systems without integrated EHRs to ensure informational continuity [10]. Although this may not be an issue for patients in single payer systems outside the U.S. or patients in integrated U.S.-based health systems (e.g., Kaiser Permanente), fragmented care is a common problem for many U.S. patients.

To understand the reasons why veterans attain increasing numbers of VA prescribers, we conducted focus groups with a subset of veterans included in the aforementioned retrospective claims-based analysis [2]. We were interested in veterans’ experiences with having multiple prescribers of cardiometabolic medications, their perceptions of advantages and disadvantages of having multiple prescribers, and the effects of having multiple prescribers on medication supply. Results from this analysis may inform outpatient management of MCC patients.

## Methods

### Participants and setting

Participants were recruited from a cohort of veterans from a single the Durham Veterans Affairs Medical Center (VAMC). Human subjects approval was obtained from the Durham VAMC as well as Duke University Medical Center and the University of North Carolina at Chapel Hill.

**Figure 1:**
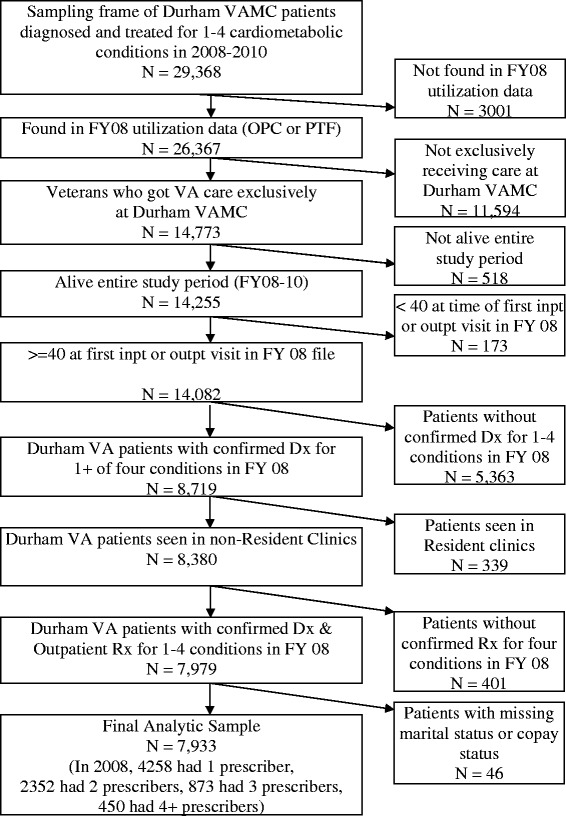
**Flow diagram for Durham VA patients.**

Eligible participants had one to four cardiometabolic conditions (i.e., hypertension, type 2 diabetes, hyperlipidemia, or congestive heart failure). We chose to focus on these conditions because they are concordant, meaning that they are related in their pathogenesis and require a similar care plan [11]. These conditions are also the most prevalent and costly chronic in the US [12,13], and associated morbidity and mortality can be reduced significantly by medication adherence. This cohort was established via electronic medical record data to address several research questions, one of which is reported herein. Details on sample selection have been reported [2]. The final study sample after exclusions (e.g., death during the study years) for the claims analyses comprised 7,933 veterans (Figure 1). From the 7,933 patients, 1,999 were randomly selected to receive a mail survey addressing other study aims (results not presented herein). From the 1,999 veterans who were sent surveys, 300 veterans (regardless of whether they were survey responders or non-responders) were randomly selected to receive a recruitment letter for this focus group study.

Patients received a recruitment letter by mail describing the study. Patients could opt out of the study by calling a toll-free telephone number. Otherwise, a project coordinator called patients one to two weeks after mailing recruitment letters. Interested and eligible patients were scheduled for a focus group discussion. Reminder calls were made the day prior to the scheduled focus group.

### Procedures

We convened three focus groups in November 2011 to solicit patients’ experiences with having multiple prescribers of cardiometabolic medications. Based on our experience and common practice, we expected that this sample size would allow us to achieved informational redundancy. We used conventional content analysis because the research goal was to describe a phenomenon when there is limited existing theory or literature [14]. In this approach, the investigators allow themes to emerge from the data instead of using a priori generated themes. Accordingly, our moderator guide comprised a short list of open-ended questions, and probes elicited further explanation from participants when necessary. Questions addressed how many and what type of physician(s) prescribe patients’ medications for cardiometabolic conditions, advantages and disadvantages of having one versus multiple prescribers of these medications, and experiences obtaining these medications inside and outside VA.

Written informed consent was obtained prior to the discussions. A social psychologist with experience in qualitative data collection and analysis (CIV) moderated the discussion, with a health economist (MLM) and project coordinator in attendance. The focus groups were digitally audio-recorded and transcribed. Participants received a meal and $25 for their time.

### Data analysis

Data analysis and collection occurred iteratively. After each focus group was conducted, the social psychologist and health economist met to discuss the results and determine if additional probing was warranted in subsequent discussions. After the third focus group, it was felt that informational redundancy was achieved, so no further data collection was conducted.

After all focus groups were conducted, the transcripts were content-analyzed by the social psychologist and health economist. Due to the backgrounds of the investigators, there could have been a heightened sensitivity regarding cost-related issues. All codes were emergent, being determined from the transcribed text rather than being generated *a priori*. These emergent codes were generated in initial readings of the transcripts by each of the coders. The codes were then refined by a systematic process of consensus among the two coders. After the final coding scheme was generated, it was applied to all transcripts by a single coder (the social psychologist), and memos were recorded during the process to reflect relationships among codes and contextual situations for the findings (e.g., VA vs. non-VA prescribers among dual users). Data were managed with Atlas.ti [15], a qualitative data analysis software program that facilitates management of coding and memo recording and analysis of patterns across transcripts. Participant demographic characteristics were obtained from the VA electronic medical record.

## Results

### Participant characteristics

Three focus groups of 6–9 veteran patients each were convened (total n = 23); analysis of the transcripts suggested informational redundancy was achieved with this sample size. Participants were aged 65 years on average (Table 1). Most participants were male (91%), Caucasian (61%), married (70%), and have to pay medication copayments in VA (70%).

**Table 1:** **Characteristics of participants (n = 23)**

Age, mean (standard deviation)	65.4 (11.1)
Male, n (%)	21 (91.3)
Race, n (%)	
Caucasian	14 (60.9)
Non-Caucasian	9 (39.1)
Marital Status, n (%)	
Married	16 (69.6)
Divorced/widowed	6 (26.1)
Never married	1 (4.3)
Must pay health care copayments, n (%)	8 (34.8)
Must pay medication copayments, n (%)	16 (69.6)
Number of conditions, mean (sd)	2.3 (0.5)
Patients diagnosed with…, n (%)	
One condition	0 (0.0)
Two conditions	16 (69.6)
Three + conditions	7 (30.4)
Patients diagnosed with…, n (%)	
Hypertension	23 (100.0)
Hyperlipidemia	19 (82.6)
Diabetes	10 (43.5)
Congestive heart failure	1 (4.3)

### Advantages of having single or multiple prescribers within VA included perceived coordination of care, lower cost, and single location

Some participants received all of their cardiometabolic medications from a single VA primary care provider (PCP), citing trust and informational continuity due to a long-standing relationship as advantages of this arrangement. Other participants had multiple VA prescribers, usually one PCP and at least one specialist but sometimes multiple primary care providers.

Participants reported that multiple prescribers arose via referrals to specialists, unavailability of usual prescribers, or lack of tenure of usual prescribers. One patient noted the advantage of being referred to a specialist:

“He (primary care doctor) knows his limitations and says, ‘I think you need to go to a different doctor, so I’ll make an appointment for you.’ So it is important that a doctor recognize his limitations.”

Whether participants had a single or multiple VA prescribers, they felt that using VA as a single source of cardiometabolic medications promoted coordinated care via the ability of providers to communicate with one another due to the presence of an electronic health record. One participant stated:

“What’s great about the VA is that everyone is on the same system. My primary care doctor can access the medicines that were prescribed for me…So that system that’s in place, that improvement, is something that lets me know they are really trying to make things better.”

Another perceived advantage of obtaining all cardiometabolic medications from VA was minimizing medication cost due to the relatively low co-payment and ability to obtain 90-day supplies.

### Disadvantages of having single or multiple prescribers within VA included lack of timeliness of obtaining refills and insufficient access to medications and prescribers

Difficulty in obtaining medications was described in the context of using the telephone-based, automatic refill system. In some cases, participants were mailed medication before they believed they needed it. In other cases, participants believed that they needed a refill, whereas the system indicated that they were not due, as noted by one participant:

“I know if you’re out of sync--you order it late and it expires--you know, they won’t give it to you. They may give you a five-day or ten-day supply to get your primary doctor to come back and set a new prescription up.”

Participants also reported that they could not schedule an appointment with their PCP to obtain refills on an emergent basis so sought Emergency Department care to obtain refills. One participant described his experience:

“He said, ‘Well, we can get you in January.’ I was supposed to be in August, and my medicine’s about ready to run out, so I am going to have to run to the emergency room today and get another series of medicines in order to cover me until January.”

### Advantages of having multiple prescribers within and outside VA included reduced cost, access to restricted medications, and convenience

Patients actively sought medications from multiple prescribers within and outside VA if they had non-VA coverage and non-VA sources could provide medication at lower cost. As one participant noted:

“(Retailer) has a big long list of medicines that they can give you for $10 a month. Two months is $6, three months is $10. And some of the medicines that I take, I can get them at (Retailer) for $10 instead of $27. That makes a big difference, and why do I want to come to the VA doctor and pay $27 for it when I can get it for $10?”

However, patients were willing to pay more for medications not on the VA formulary, which were perceived to be more efficacious. As exemplified by one participant:

“My primary writes my prescriptions, some of them to (Retailer) because he can get a better drug for me at (Retailer) than he can from the VA.”

Convenience was another perceived advantage of using non-VA prescribers because veterans were able to obtain prescriptions on an urgent basis rather than waiting for several weeks for an appointment in VA. Participants referred to having non-VA prescribers and prescriptions at non-VA pharmacies as having a “back-up.” In some cases, participants saw an outside prescriber due to convenience but had these non-VA prescriptions filled in VA to save money.

### Disadvantages of having multiple prescribers within and outside VA included less coordinated care and not accepting prescriptions from outside prescribers

One negative consequence of using non-VA providers to obtain medications was that participants felt responsible for coordinating care by providing self-management data, prescriptions, and test results to both VA and non-VA prescribers to ensure informational and management continuity. One participant stated:

“You got to be responsible. They don’t really talk to each other like that. I don’t even think the doctors in the VA talk to each other like that.”

Another disadvantage of seeing non-VA providers was that some VA physicians would not accept prescriptions written by non-VA providers. Participants cited instances of VA providers wanting to see the patient again or ordering tests that participants already had, which added inconvenience and cost. As one participant described:

“I used to use two primaries, a civilian primary and a VA primary. I’ve found it causes conflicts of interest; this one will say this and this one will say that… This one said, “You don’t need all this,” and this one said, “You do need all this.” Somebody’s gotta go, so I dropped the civilian one, and everything is flowing just like it’s supposed to.”

## Discussion

Continuity of care is assumed to be maximized when patients obtain all their care from a single provider or healthcare team [10]. Some focus group participants obtained all of their cardiometabolic medications from within VA and perceived this arrangement as beneficial. One perceived benefit of this arrangement is the low VA co-payment for medications, which was expected since non-VA medication copayments have increased markedly in the past decade [16] and cost-related non-adherence to essential medications is a significant problem [17]. Additionally, participants assumed that the EHR facilitated communication between providers and thus promoted continuity of care. This finding is consistent with previous literature indicating that patients do not observe coordination of care, but infer it from the absence of problems [4].

Although obtaining care from a single health care provider within VA was perceived as beneficial by some participants, other participants reported several benefits of having both VA and non-VA prescribers. Patients with dual coverage often preferred to use VA and non-VA care sources to enhance timeliness of obtaining medications, provide access to medications not on the VA formulary, and minimize out-of-pocket medication costs. This strategic selection of VA or non-VA prescribers was evidenced because most veterans were aware of whether VA copayments for each of their medications were higher or lower than non-VA medications. Having multiple prescribers also provided participants more flexibility in scheduling appointments on an emergent basis.

Despite these advantages, patients with VA and non-VA prescribers had to play an active role in coordinating their care by transferring information between VA and non-VA prescribers. Previous research indicates that many patients prefer to have an active role in coordinating their care [4] and that more activated patients have less adverse outcomes [3]. Thus, to the extent that veterans are actively involved in their care, seeking multiple prescribers from within and outside VA health care system may not compromise informational or management continuity or their outcomes.

On the other hand, some patients may be unwilling or unable to coordinate their care, especially if they have lower literacy or familiarity with the healthcare system [4]. Certainly, some participants reported being unable to effectively navigate the VA pharmacy system, obtain a gap appointment to have medications refilled, or contact their VA prescribers between appointments. These patients sometimes had to go to the Emergency Department to have their medications refilled. For such patients, having multiple VA prescribers may be associated with adverse outcomes, as we found in our previous claims-based analyses [2,6]. To minimize these adverse patient outcomes, interventions are needed to help transfer information within and between health care systems independent of active patient engagement.

Previous research with MCC patients indicates that patient care goals may be at odds with provider care goals. We found this to be true in the context of obtaining medications for concordant chronic conditions. Whereas patients perceived prescriber priorities to be minimizing the number of cooks in the kitchen (e.g., by not accepting prescriptions from outside providers) and centralizing prescriptions in one location (namely, VA), patients’ priorities were focused on increasing access/convenience and reducing cost, even if that meant having more cooks in the kitchen. This suggests that prescribers of medications for MCC patients should engage in open discussion with patients about their priorities. Prescribers may be able to help minimize negative outcomes by helping patients develop a detailed plan for refilling their medications before they expire and helping patients problem solve about how to obtain medications if that plan fails.

A unique feature of the VA health care system is that there are no financial benefits to providers or to VA for utilizing specific medications. Thus, prescribers should discuss with patients strategies for obtaining generic medications outside VA when the co-payment is lower; many VA prescribers do this at present. An information sheet could be provided that lists VA medications and co-payments to enable quick comparison to medications available at retail pharmacies.

Due to the qualitative methods used in this study, we cannot infer prevalence of patient experiences or attitudes. The goal of qualitative inquiry is to “sample for possibilities.” [18] Accordingly, findings that were generated by only one participant were considered as important as findings generated by multiple participants. We obtained theme saturation by the third focus group, indicating that we had sampled perspectives from veterans who were willing to participate in this study. It is important to recognize that the patients who agreed to participate in one of our focus groups may be relatively more activated and not represent experiences of less activated patients. Additionally, most participants indicated dual eligibility by their ability to obtain care from non-VA providers, so these findings may not fully reflect those of veterans who only have VA coverage. Our findings were obtained from a predominantly male sample recruited from a single VA medical center and may not generalize to other populations or settings, such as patients in single-payer health systems or uninsured patients. Future research should examine whether similar findings occur among patients with discordant conditions (i.e., that have different pathogenesis and require different care plans).

## Conclusions

The Agency for Healthcare Research & Quality indicates that “patients are recognized as potentially the only common thread linking interdependent clinicians and settings and may represent the only perspective and data source from which coordination of care may be measured” [19]. Our findings suggest that many patients with multiple, concordant conditions actively seek multiple prescribers of medications for their chronic conditions to maximize access and convenience as well as minimize cost. Given that increasing numbers of prescribers is associated with worse refill adherence and health care utilization outcomes [2,6], interventions should create an open dialogue between patients and prescribers to create a shared understanding of goals and priorities for the health care plan in general and chronic disease medications in particular. Such interventions should be evaluated for their potential to reduce patient medication nonadherence and healthcare utilization and improve health outcomes such as disease control.

## References

[1] Pham HH, Schrag D, O’Malley AS, Wu B, Bach PB (2007). Care patterns in Medicare and their implications for pay for performance. N Engl J Med.

[2] Maciejewski ML, Powers BJ, Sanders L, Farley JF, Hansen RA, Sleath B, Voils CI (2014). The intersection of patient complexity, prescriber continuity and acute care utilization. J Gen Intern Med.

[3] Maeng DD, Martsolf GR, Scanlon DP, Christianson JB (2012). Care coordination for the chronically ill: Understanding the patient’s perspective. Health Serv Res.

[4] Haggerty JL, Roberge D, Freeman GK, Beaulieu C (2013). Experienced continuity of care when patients see multiple clinicians: a qualitative metasummary. Ann Fam Med.

[5] Reid RJ, Fishman PA, Yu O, Ross TR, Tufano JT, Soman MP, Larson EB (2009). Patient-centered medical home demonstration: A prospective, quasi-experimental, before and after evaluation. Am J Manag Care.

[6] Hansen RA, Powers BJ, Sanders L, Farley JF, Voils CI, Sleath B, Maciejewski ML: **Prescriber continuity and medication adherence for complex patients.** in pres. 10.1177/1060028014563266 25549627

[7] Steinman MA, Lee SJ, John Boscardin W, Miao Y, Fung KZ, Moore KL, Schwartz JB (2012). Patterns of multimorbidity in elderly veterans. J Am Geriatr Soc.

[8] Yoon J, Zulman D, Scott JY, Maciejewski ML (2014). Costs associated with multimorbidity among VA Patients. Med Care.

[9] Liu CF, Manning WG, Burgess JF, Hebert PL, Bryson CL, Fortney J, Perkins M, Sharp ND, Maciejewski ML (2011). Reliance on Veterans Affairs outpatient care by Medicare-eligible veterans. Med Care.

[10] Saultz JW (2003). Defining and measuring interpersonal continuity of care. Ann Fam Med.

[11] Piette JD, Kerr EA (2006). The impact of comorbid chronic conditions on diabetes care. Diabetes Care.

[12] **Chronic conditions: making the case for ongoing care.** [http://www.rwjf.org/content/dam/farm/reports/reports/2010/rwjf54583]

[13] Centers for Medicare and Medicaid Services (2012). Chronic Conditions among Medicare Beneficiaries.

[14] Hsieh H-F, Shannon SE (2005). Three approaches to qualitative content analysis. Qual Health Res.

[15] Dowling M (2008). ATLAS.ti (Software). The SAGE Encyclopedia of Qualitative Research Methods. SAGE Publications, Inc.

[16] Claxton G, Rae M, Panchal N, Whitmore H, Damico A, Kenward K (2014). Health Benefits In 2014: Stability In Premiums And Coverage For Employer-Sponsored Plans. Health Aff.

[17] Goldman DP, Joyce GF, Escarce JJ, Pace JE, Solomon MD, Laouri M, Landsman PB, Teutsch SM (2004). Pharmacy benefits and the use of drugs by the chronically ill. JAMA.

[18] Wood M, Christy R (1999). Sampling for possibilities. Qual Quant.

[19] McDonald K, Sundaram V, Bravata D (2007). Closing the quality gap: A critical analysis of quality improvement strategies. Conceptual frameworks and their application to evaluating care coordination interventions.

